# Alpha fetoprotein antagonizes apoptosis induced by paclitaxel in hepatoma cells *in vitro*

**DOI:** 10.1038/srep26472

**Published:** 2016-06-03

**Authors:** Mingyue Zhu, Wei Li, Yan Lu, Xu Dong, Yi Chen, Bo Lin, Xieju Xie, Junli Guo, Mengsen Li

**Affiliations:** 1 Hainan Provincial Key Laboratory of Carcinogenesis and Intervention, Hainan Medical College, Haikou 571199, P.R. China; 2 Key Laboratory of Molecular Biology, Hainan Medical College, Haikou 571159, P.R. China; 3 Department of Pathophysiology, Hainan Medical College, Haikou 571199, China; 4 Institution of Tumours, Hainan Medical College, Haikou 570102, P.R. China

## Abstract

Hepatocellular carcinoma (HCC) cell resistance to the effects of paclitaxel has not been adequately addressed. In this study, we found that paclitaxel significantly inhibited the viability of HLE, Bel 7402 and L-02 cells in a dose- and time-dependent manner. HLE cells and L-02 cells resisted the cytotoxicity of paclitaxel when transfected with pcDNA3.1-*afp* vectors. However, Bel 7402 cell sensitivity to paclitaxel was increased when transfected with alpha fetoprotein (AFP)-siRNA. Bel 7402 cell resistance to paclitaxel was associated with the expression of the “stemness” markers CD44 and CD133. Paclitaxel significantly inhibited growth and promoted apoptosis in HLE cells and L-02 cells by inducing fragmentation of caspase-3 and inhibiting the expression of Ras and Survivin, but pcDNA3.1-*afp* vectors prevented these effects. However, paclitaxel could not significantly promote the cleavage of caspase-3 or suppress the expression of Ras and Survivin in Bel 7402 cells. Silenced expression of AFP may be synergistic with paclitaxel to restrain proliferation and induce apoptosis, enhance cleavage of caspase-3, and suppress the expression of Ras and Survivin. Taken together, AFP may be an important molecule acting against paclitaxel-inhibited proliferation and induced apoptosis in HCC cells via repressing the activity of caspase-3 and stimulating the expression of Ras and Survivin. Targeted inhibition of AFP expression after treatment with paclitaxel is an available strategy for the therapy of patients with HCC.

Paclitaxel is an anticancer drug originally derived from the pacific yew tree (Taxus brevifolia). It stabilizes microtubules and inhibits depolymerization back to tubulin, resulting in mitotic inhibition. Such an effect causes cell cycle arrest in the G2/M phase and induces cell death through an apoptotic pathway^1^,^2^. Paclitaxel is now widely used as an effective chemotherapeutic agent for the treatment of common cancers, such as those of the breast, lungs and ovaries^3^. Hepatocellular carcinoma (HCC) is one of the most prevalent cancers and many patients develop either unresectable or metastatic disease. Surgery is considered the best method for HCC therapy, but unfortunately a majority of patients with HCC are not suitable for surgery at diagnosis. The survival ratio of HCC patients is very low because HCC cells are less sensitive or become resistant to anti-cancer drugs after consecutive therapy. There is an urgent need to explore the mechanism of HCC resistance to chemotherapy and to develop new approaches to cure drug-resistant HCC patients.

Alpha fetoprotein (AFP) is an early biomarker for the diagnosis of HCC. High levels of serum AFP are closely associated with the malignant behaviour of HCC cells^4^,^5^,^6^. Many researchers have found that AFP is anti-apoptotic^7^,^8^ and plays an important role in promoting proliferation^9^ and resisting the cytotoxicity of 5-Fluorouracil (5-Fu) and all *trans* retinoic acid (ATRA)^10^,^11^,^12^,^13^,^14^ and other drugs, such as tumour necrosis factor-related apoptosis induced-ligand (TRAIL), in HCC cells^15^. Recently, we have found that AFP suppressed the transduction of the ATRA receptor signal to antagonize the apoptosis induced by ATRA^13^,^14^. This evidence suggested that the expression of AFP is a pivotal factor involved in drug resistance in HCC cells, and AFP plays a role in suppressing lymphocyte-induced apoptosis in HCC cells^15^. Clinical trials have indicated that whe ther the expression of AFP plays a role in HCC resistance to paclitaxel^16^,^17^ is unclear. In this study, we found that the expression of AFP in HCC cells was a pivotal cytoplasmic molecule for the resistance to paclitaxel of HCC cells *in vitro*.

## Results

### Paclitaxel inhibited proliferation of HCC cells and human normal liver cells

**Figure 1:**
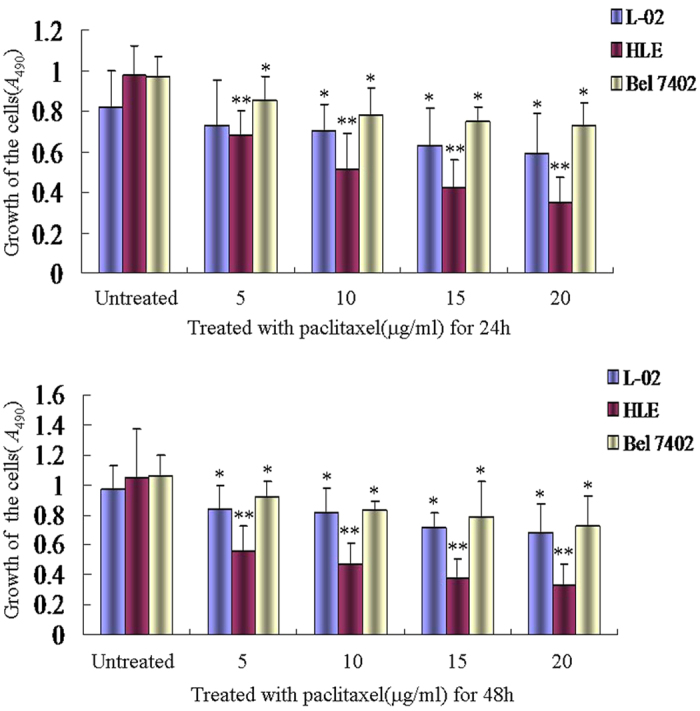
Effects of paclitaxel on the growth of human normal liver cells and human hepatoma cells *in vitro*. The human normal liver cell line, L-02 cells, and human hepatoma cell lines, HLE and Bel 7402 cells, were treated with paclitaxel at concentrations of 5 μg/ml, 10 μg/ml, 15 μg/ml and 20 μg/ml for 24 hrs and 48 hrs. The growth of the cells was analyzed by MTT. *P < 0.05 and **P < 0.01 vs untreated groups. N = 6.

The MTT results indicated that in the HCC cells, Bel 7402 and HLE, human normal liver cells, L-02 treated with different concentrations of paclitaxel (5–20 μg/ml), the growth of these cells was significantly inhibited when paclitaxel was present at >10 μg/ml. Interestingly, the growth of HLE cells was significantly suppressed compared to Bel 7402 cells and L-02 cells (Fig. 1). This result indicated that paclitaxel was able to inhibit the growth of HCC cells and normal liver cells.

### AFP antagonized growth inhibited by paclitaxel

**Figure 2:**
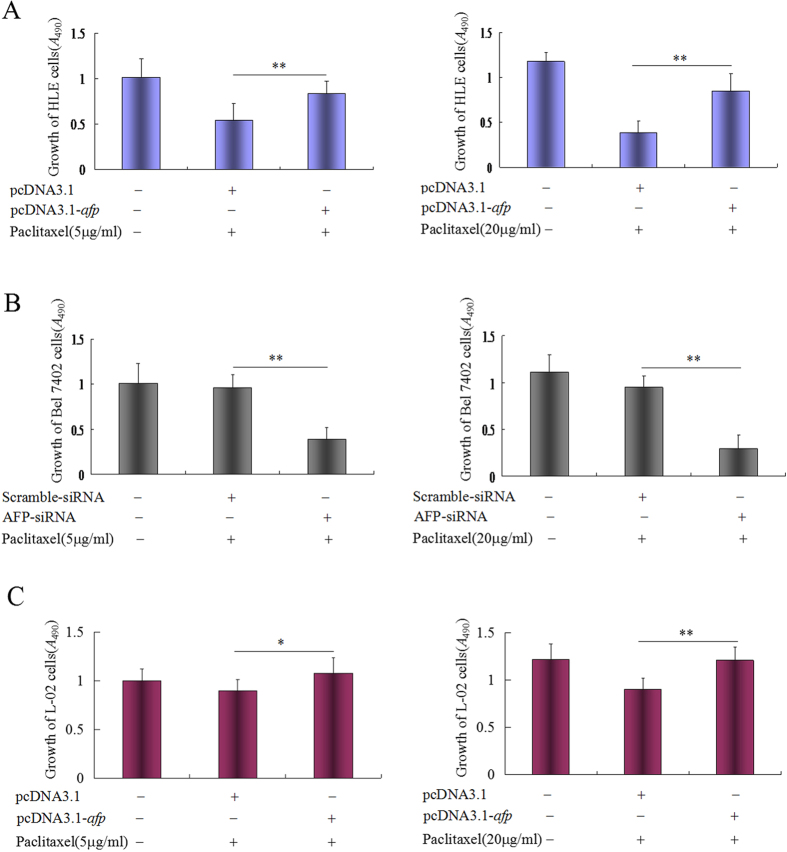
Effects of AFP on paclitaxel inhibition of the growth of the human hepatoma cell lines, HLE and Bel 7402, and human normal liver cell line L-02 *in vitro*. (**A**) HLE cells were transfected with pcDNA3.1-*afp* vectors for 24 hrs followed by treatment with paclitaxel at concentrations of 5 μg/ml and 20 μg/ml for 24 hrs, respectively. The growth of HLE cells was detected by MTT. **P < 0.01, N = 6. (**B**) Bel 7402 cells were transfected with AFP-siRNA vectors for 24 hrs followed by treatment with paclitaxel at concentrations of 5 μg/ml and 20 μg/ml for 24 hrs, respectively. The growth of HLE cells was detected by MTT. **P < 0.01, N = 6. (**C**) L-02 cells were transfected with pcDNA3.1-*afp* vectors for 24 hrs followed by treatment with paclitaxel at concentrations of 5 μg/ml and 20 μg/ml for 24 hrs. The growth of L-02 cells was detected by MTT. **P < 0.05, **P < 0.01, N = 6.

To explore the effect of AFP on paclitaxel in regulating the proliferation of HCC cells and normal liver cells, Bel 7402 cells were transfected with AFP-siRNA vectors, and HLE or L-02 cells were transfected with pcDNA3.1-*afp* vectors followed by treatment with paclitaxel (5 μg/ml and 20 μg/ml). MTT analysis indicated that the sensitivity to paclitaxel was restrained in HLE cells transfected with pcDNA3.1-*afp* vectors (Fig. 2A). However, silenced expression of AFP increased the sensitivity to paclitaxel in Bel 7402 cells (Fig. 2B). The sensitivity to paclitaxel was also inhibited in L-02 cells while transfected with pcDNA3.1-*afp* vectors (Fig. 2C). These results showed that AFP is antagonistic to paclitaxel, inhibiting the proliferation of HCC cells and normal liver cells.

### AFP regulated paclitaxel-induced apoptosis in HCC cells and normal liver cells

**Figure 3:**
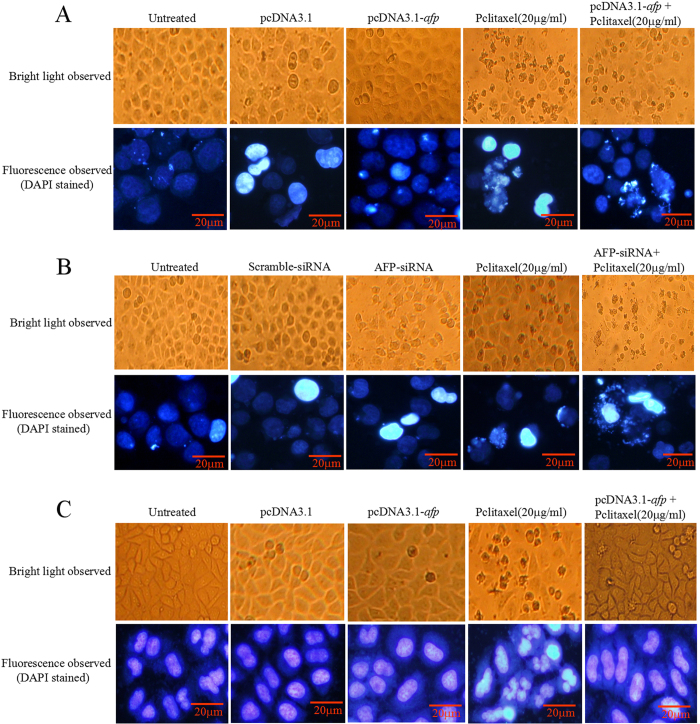
Effects of AFP on paclitaxel inducing apoptosomes in human hepatoma cells and L-02 cells *in vitro*. (**A**) HLE cells were treated with paclitaxel at a concentration of 20 μg/ml and transfected with pcDNA3.1-*afp* vectors for 24 hrs followed by treatment with paclitaxel (20 μg/ml) for 24 hrs. The morphological changes in HLE cells were observed by microscopy. The cytoblasts of HLE cells were stained with DAPI and observed by fluorescent microscopy. B, Bel 7402 cells were treated with paclitaxel at a concentration of 20 μg/ml and transfected with AFP-siRNA vector for 24 hrs followed by treatment with paclitaxel (20 μg/ml) for 24 hrs. The morphological changes in Bel 7402 cells were observed by microscopy. The cytoblasts of Bel 7402 cells were stained with DAPI and observed by fluorescent microscopy. C, L-02 cells were treated with paclitaxel at a concentration of 20 μg/ml and transfected with pcDNA3.1-*afp* vectors for 24 hrs followed by treatment with paclitaxel (20 μg/ml) for 24 hrs. The morphological changes in L-02 cells were observed by microscopy. The cytoblasts of L-02 cells were stained with DAPI and observed by fluorescent microscopy. The images were representative of at least three independent experiments.

**Figure 4:**
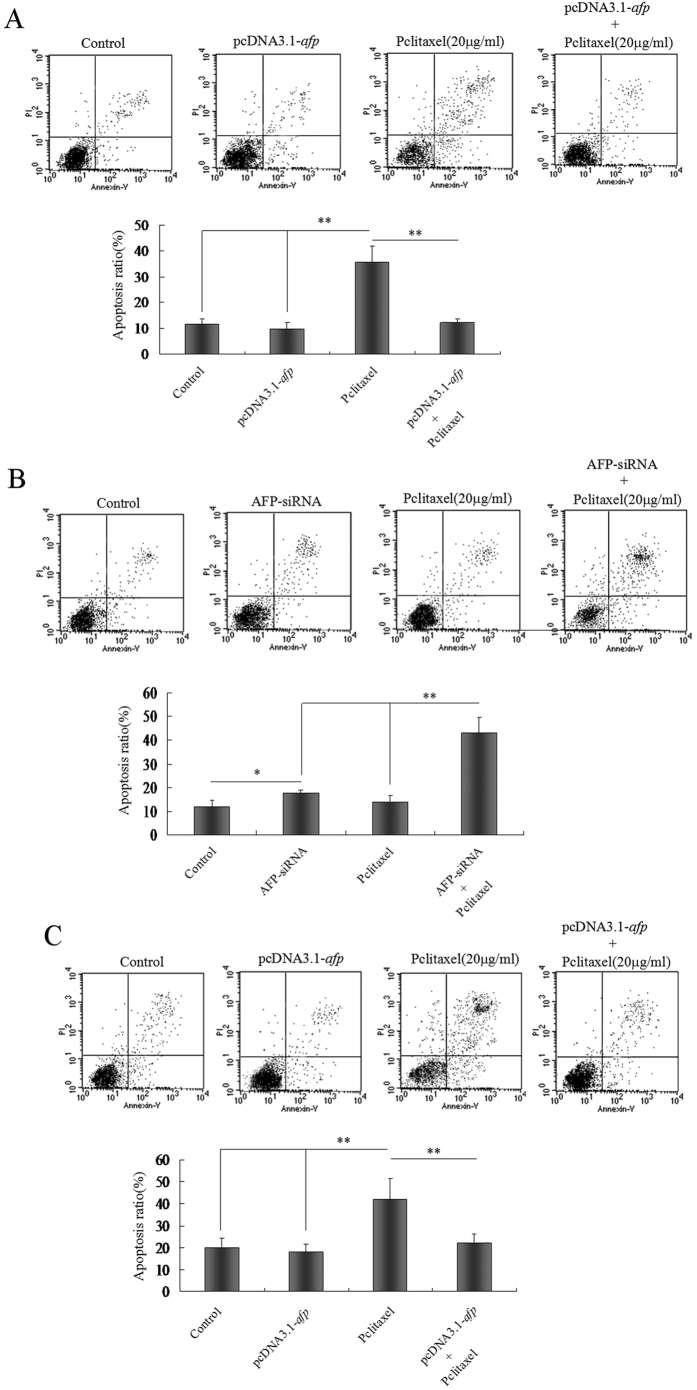
Effects of AFP on apoptosis induced by paclitaxel in hepatoma cells and L-02 cells. (**A**) HLE cells were transfected with pcDNA-*afp* vectors for 24 hrs followed by treatment with paclitaxel (20 μg/ml) for 24 hrs. Apoptosis in HLE cells was detected by flow cytometry. The low columnar picture is the statistical analysis of the apoptosis ratio. **P < 0.01; (**B**) Bel 7402 cells were transfected with AFP-siRNA for 24 hrs followed by treatment with paclitaxel (20 μg/ml) for 24 hrs. Apoptosis in Bel 7402 cells was detected by flow cytometry. The low columnar picture is the statistical analysis of the apoptosis ratio. *P < 0.05; **P < 0.01. (**C**) L-02 cells were transfected with pcDNA-*afp* vectors for 24 hrs followed by treatment with paclitaxel (20 μg/ml) for 24 hrs. Apoptosis in L-02 cells was detected by flow cytometry. The low columnar picture is the statistical analysis of the apoptosis ratio. **P < 0.01. The images were representative of at least three independent experiments.

The previous results suggested that AFP could antagonize paclitaxel-inhibited growth of HCC cells. However, whether AFP played a role in paclitaxel suppression of apoptosis in HCC cells and normal liver cells remained unclear. In this investigation, we examined whether paclitaxel promoted the generation of the apoptosome and apoptosis in HCC cells and normal liver cells by microscopy and flow cytometric analysis, respectively. Microscopic observation indicated that the apoptosome number was significantly decreased in HLE cells transfected with pcDNA3.1-*afp* vectors following treatment with paclitaxel compared to pcDNA3.1-*afp,* control vectors and untreated groups (Fig. 3A). However, the apoptosome number was significantly increased in Bel 7402 cells transfected with AFP-siRNA vectors following treatment with paclitaxel compared to AFP-siRNA vectors, control vectors and untreated groups (Fig. 3B). The apoptosome number was also significantly decreased in L-02 cells transfected with pcDNA3.1-*afp* (Fig. 3C). The flow cytometric analysis results also revealed that the number of apoptotic HLE and L-02 cells was significantly decreased in cells transfected with pcDNA3.1-*afp* vectors following treatment with paclitaxel than in pcDNA3.1-*afp* vectors, control vectors and untreated groups (Fig. 4A,C). However, the number of apoptotic Bel 7402 cells was significantly higher when transfected with AFP-siRNA vectors following treatment with paclitaxel than in AFP-siRNA vectors, control vectors and untreated groups (Fig. 4B). These results demonstrated that AFP plays a pivotal role in confronting paclitaxel-induced apoptosis in HCC cells.

### Stemness cells traits were merged in Bel 7402 cells resistant to paclitaxel

**Figure 5:**
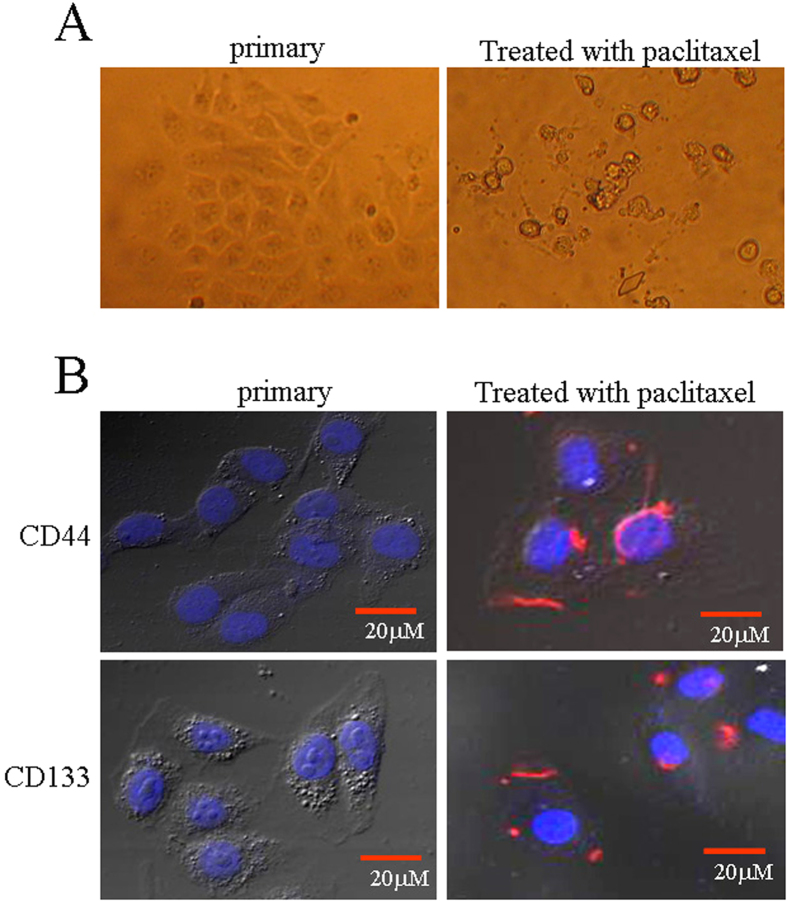
Expression of stemness markers in paclitaxel-resistant Bel 7402 cells. (**A**) Bel 7402 cells were treated with paclitaxel (20 μg/ml) for 48 hrs, and the morphologic changes in the cells were observed by bright-light microscopy. (**B**) Bel 7402 cells were consecutively treated with paclitaxel (20 μg/ml) for 7 d, and the cells were washed with PBS three times. The expression of the stemness markers CD44 and CD133 in the cells was observed by laser confocal microscopy; the red staining shows the location of the markers. The images are representative of at least three independent experiments.

Although the results indicated that HCC cells and normal liver cells are sensitive to paclitaxel, a small number of Bel 7402 cells were resistant to the cytotoxicity of paclitaxel. Because Bel 7402 cells are a HCC cell line with high expression of AFP, in this investigation, we detected stem cell markers in the cells which resistant to paclitaxel. Light microscopy observation revealed the change in apoptotic morphology of Bel 7402 cells (Fig. 5A), and laser confocal microscopy was used to observe the expression and location of stemness markers in the cells. The results indicated that the stemness markers CD33 and CD144 were highly expressed and located in the membrane of the cells (Fig. 5B). These results suggested that the resistance of Bel 7402 cells to paclitaxel cytotoxicity might be related to the generation of stem cells.

### AFP restrained caspase-3 activity and promoted the expression of Ras and survivin to antagonize paclitaxel-induced apoptosis in HCC cells and normal liver cells

**Figure 6:**
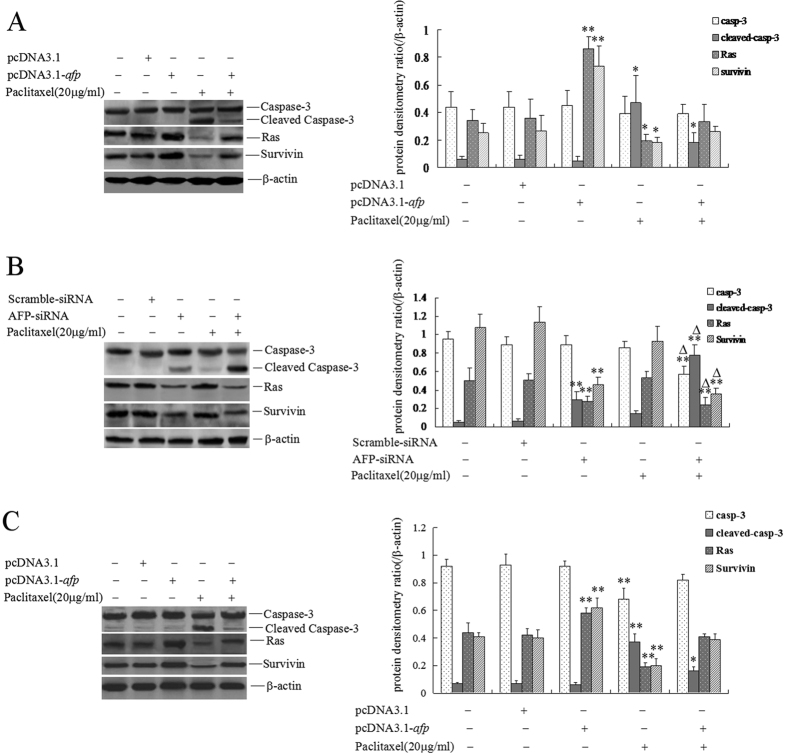
Effects of AFP and paclitaxel on the expression of caspase-3, Ras and Survivin in hepatoma cells and L-02 cells. (**A**) HLE cells were treated with paclitaxel at a concentration of 20 μg/ml and transfected with pcDNA3.1-*afp* vectors for 24 hrs. Followed treatment with paclitaxel (20 μg/ml) for 24 hrs, the expression of caspase-3, cleaved caspase-3, Ras and survivin in the cells was detected by Western blotting. The right columnar images represent the protein densitometry values compared to the internal control β-actin ratio. *P <  0.05 vs untreated groups, pcDNA3.1 treated group and pcDNA3.1-*afp* treated groups; **P < 0.01 vs untreated groups, pcDNA3.1 treated group and pcDNA3.1-*afp* and paclitaxel co-treated groups. (**B**) Bel 7402 cells were treated with paclitaxel at a concentration of 20 μg/ml and transfected with AFP-siRNA vectors for 24 hrs. Following treatment with paclitaxel (20 μg/ml) for 24 hrs, the expression of caspase-3, cleaved caspase-3, Ras and survivin in the cells was detected by Western blotting. The right columnar images represent the protein densitometry values compared to the internal control β-actin ratio. **P < 0.01 vs untreated groups, scramble-siRNA treated group and paclitaxel treated groups; *P < 0.05 vs AFP-siRNA treated groups, AFP-siRNA and paclitaxel co-treated groups. (**C**) L-02 cells were treated with paclitaxel at a concentration of 20 μg/ml and transfected with pcDNA3.1-*afp* vectors for 24 hrs. Following treatment with paclitaxel (20 μg/ml) for 24 hrs, the expression of caspase-3, cleaved caspase-3, Ras and Survivin in the cells was detected by Western blotting. The right columnar images represent the protein densitometry values compared to the internal control β-actin ratio. *P < 0.05 vs untreated groups, pcDNA3.1 treated group and pcDNA3.1-*afp* treated groups; **P < 0.01 vs untreated groups, pcDNA3.1 treated group and pcDNA3.1-*afp* and paclitaxel co-treated groups. The images are a representation of at least three independent experiments.

In this study, we found that paclitaxel suppressed growth and induced apoptosis in HCC cells and that AFP played an important role in antagonizing the effect of paclitaxel. To explore the mechanism of AFP in antagonizing the effect of paclitaxel, we analyzed the expression of apoptosis-related proteins such as caspase-3, survivin and the proliferation-related protein Ras. The results showed that pcDNA3.1-*afp* vectors inhibited the cleavage of caspase-3 and promoted the expression of survivin and Ras. Paclitaxel stimulated the cleavage of caspase-3 and inhibited the expression of survivin and Ras in HLE and L-02 cells, but the effect was significantly antagonized when the cells were transfected with pcDNA3.1-*afp* vectors (Fig. 6A,C). Silenced expression of AFP by AFP-siRNA significantly induced cleavage of caspase-3 and suppressed the expression of survivin and Ras in Bel 7402 cells. The paclitaxel-induced cleavage of caspase-3 and suppression of the expression of survivin and Ras in Bel 7402 cells was not a large effect. However, silenced expression of AFP might be synergistic with paclitaxel to induce the fragmentation of caspase-3 and suppress the expression of survivin and Ras in Bel 7402 cells (Fig. 6B). These results implied that AFP antagonized the effect of paclitaxel through restraining caspase-3 activity and enhancing the expression of survivin and Ras.

## Discussion

Drug resistance in HCC cells is an urgent issue needing resolution for successful clinical therapy. Although paclitaxel is a common chemodrug for therapy of HCC patients^18^,^19^,^20^, resistance to paclitaxel in HCC patients often occurs^20^,^21^ due to inherent or acquired drug resistance in the HCC cells. The literature indicates that paclitaxel induces the apoptosis of cancer cells through arresting the M/G2 cell cycle and stimulating caspase signalling^22^,^23^. In this study, the results indicated that paclitaxel inhibited growth not only of HCC cells but also of normal liver cells (L-02). Interestingly, the effect of paclitaxel on suppressing proliferation in HLE cells (non AFP-producer) was significantly higher than in Bel 7402 cells (AFP-producer) and L-02 cells. When HLE or L-02 cells were transfected with AFP-expressed vectors, the sensitivity of HLE or L-02 cells to paclitaxel was significantly decreased and sensitivity of Bel 7402 cells was significantly enhanced after transfection with silenced AFP vectors (AFP-siRNA). These results implied that paclitaxel plays a role in restraining the growth of HCC cells. In combination with differential sensitivity to paclitaxel in HCC cells, AFP may play an important role in antagonizing the effect of paclitaxel-inhibited proliferation of HCC cells.

AFP is a diagnostic cancer marker of HCC occurrence. Many researchers have demonstrated that AFP is closely related to malignant behaviour, including resistance to drug cytotoxicity in HCC cells. Chemotherapy is the most common treatment option for various cancers, and chemicals inhibit cancer cell growth through inducing apoptosis of the cancer cells. Paclitaxel therapy of HCC patients may promote apoptosis in cancer cells. In this study, we found that paclitaxel induces the generation of the apoptosome in HLE cells, Bel 7402 cells and L-02 cells, and promotes apoptosis in these cells. Overexpression of AFP in HLE or L-02 cells confronted the effect of paclitaxel; However, silenced expression of AFP in Bel 7402 cells increased the effect of paclitaxel. Previously, we have found that AFP inhibits the transcriptional activity of retinoic acid receptor-β(RAR-β) leading to Bel 7402 cell resistance to apoptosis induced by ATRA and TRAIL^11^,^15^. These results suggested that paclitaxel induced apoptosis in HCC cells and that AFP antagonises paclitaxel-promoted apoptosis of HCC cells.

The literature shows that cancers originate from cancer stem cells (CSCs)^24^,^25^,^26^. CSCs are a pivotal factor for cancer cell resistance to chemotherapy and metastasis^27^,^28^,^29^,^30^. Epithelial cell adhesion molecule (EpCAM)(+)/AFP(+) HCC cells have enriched hepatic CSCs. In previous study, targeted EpCAM inhibited the initiation of CSC populations in human HCCs^31^. In the present study, we selected Bel 7402 cells that expressed AFP to demonstrate that AFP was associated with the origination of CSCs and resistance to paclitaxel. CD44 and CD133 are authentic markers of stem cells and the expression of these markers suggested the generation of stem cells. In this study, we screened for Bel 7402 cells resistant to paclitaxel and detected the expression of CD44 and CD133. The results revealed a high expression of CD44 and CD133 in the cellular membrane in paclitaxel-resistant Bel 7402 cells, but the expression of CD44 and CD133 could not merge in primary Bel 7402 cells. AFP also acts as a stem cell marker in liver stem cells^32^,^33^, evidence that AFP-producing pancreatic cancer cells possess cancer stem cell characteristics^34^. Our previous study demonstrated that AFP was able to induce the expression of CD44 and CD133 in HCC cells (results not included in this paper). These results further proved that AFP-promoted generation of CSCs was a pivotal factor for Bel 7402 resistance to paclitaxel.

Cleaved caspase-3 is the active form of caspase-3. Activated caspase-3 enters into the nucleus to activate poly(ADP-ribose) polymerase 1 (PARP-1), and PARP-1 cuts off the chromosome leading to cell apoptosis^35^,^36^. Survivin plays a role in confronting chemicals and bio-drugs to induce apoptosis of cancer cells through inhibiting the transduction of the caspase cascade^37^. Ras protein could stimulate proliferation and enhance the malignant behaviours of cancer cells^38^. To explore the mechanism by which AFP antagonizes the apoptosis induced by paclitaxel in HCC cells, in this study, we detected the expression of cleaved caspase-3, Ras and survivin. The results revealed that paclitaxel was capable of stimulating cleavage of caspase-3 and suppressing the expression of Ras and survivin in HLE cells, Bel 7402 cells and L-02 cells. pcDNA3.1-*afp* vectors transfected into HLE or L-02 cells suppressed the cleavage of caspase-3 and promoted the expression of Ras and survivin, and pcDNA3.1-*afp* vectors inhibited the effect of paclitaxel in HLE or L-02 cells. However, silenced expression of AFP could increase the effect of paclitaxel in Bel 7402 cells. We have proved that the interaction of AFP with caspase-3 inhibits the transduction of the caspase signalling cascade^11^. AFP activates phosphatidylinositol 3-kinase(PI3K)/ protein kinase B(AKT) signalling to stimulate the expression of Ras and Src^39^,^40^, and paclitaxel inhibited liver metastasis of AFP-producing gastric cancer cells through restraining expression of AFP^41^. These results suggested that the expression of AFP is a critical factor for resistance to paclitaxel in HCC cells. AFP has an effect through enhancing cleavage of caspase-3 and promoting the expression of Ras and survivin. The AFP RNA-interference system provides a usable tool for HCC-specific targeted therapy^42^. Recently, we have found that AFP harbours a function for stimulating metastasis in HCC cells^43^. These results indicated that AFP was a pivotal molecule that promoted malignant behaviours in HCC cells.

In summary, the present study demonstrated that AFP plays a crucial role in acquiring drug resistance caused by paclitaxel exposure. This study provides valuable information for the improvement of chemotherapies by targeting key molecules such as AFP in paclitaxel resistant HCC patients. Importantly, the use of AFP inhibitors prior to paclitaxel treatment may be an available strategy for the therapy of HCC patients.

## Material and Methods

### Cell culture

The human HCC cell lines Bel 7402 (AFP-producer) and HLE (non-AFP-producer) were a gift of the Department of Cell Biology, Peking University Health Science Center. The L-02 cells were purchased from the Institution of Cellular Biology, Shanghai Academy of Life Science, Chinese Academy of Science. In this study, we selected these cells for testing. The cells were cultured in RPMI-1640 medium and supplemented with 10% heat-inactivated foetal calf serum (FCS). The cells were incubated at 37 °C in a humidified atmosphere containing 5% CO_2_.

### MTT methods analyzed the growth of the cells

The methylthiazolyldiphenyl-tetrazolium bromide (MTT) method was described previously^14^. Briefly, 1.5 × 10^4^ cells per well of L-02 cells, Bel 7402 cells or HLE cells were plated into 96-well plates and cultured in RPMI-1640 medium supplemented with 10% FCS at 37 °C in a humidified atmosphere of 5% CO_2_ for 48 hrs. The cultured cells were replaced with medium without FCS for another 24 hrs, and the cells were treated with different concentrations of paclitaxel (5–20 μg/ml) for 24 hrs. Bel 7402 cells were transfected with AFP-siRNA vectors, and HLE cells and L-02 cells were transfected with AFP-expressed vectors (pcDNA3.1-*afp*) for 24 hrs, respectively, followed by treatment with different concentrations of paclitaxel (5 μg/ml or 20 μg/ml) for 24 hrs. The effects of AFP on paclitaxel (Sigma, USA) regulating the growth of these cells were detected by MTT assay as described in a previous study^14^ following a regular procedure. The growth of the cells was determined by optical density (*A*_490_).

### RNA interference assay, AFP-expressed vector construction and transient transfection

The AFP-siRNA vectors and AFP-expressed vectors (pcDNA3.1-*afp*) were constructed as described in a previous study^11^. Bel 7402 cells and HLE or L-02 cells were transfected with AFP-siRNA vectors and transfected with pcDNA3.1-*afp* vectors for 48 hrs, respectively, followed by treatment with paclitaxel (5 μg/ml or 20 μg/ml) for 24 hrs. The growth, apoptosis and protein expression were evaluated by other methods.

### Cell morphology was observed and nuclear was stained with DAPI

To observe alterations in cellular morphology induced by paclitaxel (20 μg/ml) and the role of AFP in the effect of paclitaxel, 2.5 × 10^4^ cells/ml of Bel 7402, HLE or L-02 cells were plated in 6-well plates in serum-free medium for 24 hrs. The supernatant was replaced with RPMI-1640 medium supplemented with 10% FCS for 24 hrs. Bel 7402 cells were transfected with AFP-siRNA vectors, and HLE and L-02 cells were transfected with pcDNA-*afp* vectors for 24 hrs, followed by treatment with paclitaxel (20 μg/ml) for 48 hrs. Cellular morphology was observed under a microscope. The cells were stained with a 4,6-diamidino-2-phenylindole dihydrochloride (DAPI) solution. The cells were imaged using a fluorescence microscope at 400x magnification. In this study, nuclear pyknosis and fragmentation were taken to define apoptosis, and these criteria were evaluated microscopically as described in previous reports^13^,^14^.

### Flow cytometric analysis

Bel 7402 cells, HLE cells and L-02 cells were cultured in RPMI-1640 medium supplemented with 10% FCS at 37 °C in a humidified atmosphere with 5% CO_2_. Bel 7402 cells were transfected with AFP-siRNA vectors, and HLE cells and L-02 cells were transfected with pcDNA3.1-*afp* vectors for 24 hrs followed by treatment with paclitaxel (20 μg/ml) for 48 hrs. The apoptosis of Bel 7402 cells, HLE cells and L-02 cells were analyzed by flow cytometry. The detailed procedures were described in a previous study^14^.

### Laser confocal microscopy was used to observe the expression of stemness markers

Bel 7402 cells were cultured in RPMI-1640 medium supplemented with 10% FCS at 37 °C in a humidified atmosphere with 5% CO_2_ in 6-well cultured plates, and the cells were treated with paclitaxel (20 μg/ml) consecutively for 7 days. Then, the cells were washed with RPMI-1640 medium. The surviving cells and primary cells were incubated with rabbit anti-human primary antibody CD44 and CD133 (Abcam Corp. USA) for 12 hrs and incubated with TRITC-conjugated goat secondary antibody for 2 hrs. The cells were washed with PBS three times. The expression and location of CD44 and CD133 were observed by laser confocal microscopy (Fuji, Japan).

### Western blotting analysis

To estimate the influence of paclitaxel and AFP on the expression of apoptosis-related proteins, Bel 7402 cells, HLE cells and L-02 cells were treated with paclitaxel (20 μg/ml) for 48 hrs. Bel 7402 cells were transfected with AFP-siRNA vectors. HLE cells and L-02 cells were transfected with pcDNA3.1-*afp* vectors for 24 hrs followed by treatment with paclitaxel (20 μg/ml) for 48 hrs. The expression of apoptosis-related proteins such as caspase-3, cleaved caspase-3, Ras and survivin in Bel 7402 cells, HLE cells and in L-02 cells were analyzed by Western blotting. The detailed procedure was described in previous studies^14^,^15^.

### Statistical analysis

The data are presented as the mean ± S.D. Statistical analysis was performed using Student’s *t*-test for two experimental groups. Significance was set at *P* < 0.05. Statistical significance was determined using Student’s t-test and ANOVA (*F* test) (SPSS 11.5 software for Windows, SPSS Inc., Chicago, IL, US).

## Additional Information

**How to cite this article**: Zhu, M. *et al.* Alpha fetoprotein antagonizes apoptosis induced by paclitaxel in hepatoma cells *in vitro*. *Sci. Rep.* **6**, 26472; doi: 10.1038/srep26472 (2016).
